# Extracellular matrix remodelling and stiffening contributes to tumorigenesis of salivary carcinoma ex pleomorphic adenoma——A study based on patient-derived organoids

**DOI:** 10.1186/s13578-023-01071-x

**Published:** 2023-07-01

**Authors:** Wanling Chen, Ting Gu, Qianqian Chen, Chuxiang Qu, Chunye Zhang, Yuhua Hu, Ronghui Xia, Ying Zhang, Min Wang, Xinyi Huang, Jiang Li, Chaoji Shi, Zhen Tian

**Affiliations:** 1 Department of Oral Pathology, School of Medicine, Ninth People’s Hospital, Shanghai Jiao Tong University, No. 639, Manufacturing Bureau Road, Huangpu District, Shanghai, 200011 P.R. China; 2 National Center for Stomatology, National Clinical Research Center for Oral Diseases, Shanghai, 200011 China; 3 Shanghai Key Laboratory of Stomatology & Shanghai Research Institute of Stomatology, Shanghai, 200011 China; 4 Research Unit of Oral and Maxillofacial Regenerative Medicine, Chinese Academy of Medical Sciences, Shanghai, 200011 China; 5 Department of ultrasound, School of Medicine, Ninth People’s Hospital, Shanghai Jiao Tong University, Shanghai, 200011 P.R. China; 6 Department of Oral and Maxillofacial-Head Neck Oncology, Shanghai Ninth People’s Hospital, College of Stomatology, Shanghai Jiao Tong University School of Medicine, No. 639, Manufacturing Bureau Road, Huangpu District, Shanghai, 200011 China

**Keywords:** Carcinoma ex pleomorphic adenoma, Extracellular matrix, Collagen, Remodelling, Stiffness, TWIST1, Organoids, Salivary

## Abstract

**Background:**

Salivary carcinoma ex pleomorphic adenoma (CXPA) is defined as a carcinoma that develops from benign pleomorphic adenoma (PA). Abnormally activated Androgen signaling pathway and amplification of HER-2/*neu(ERBB-2)* gene are known to be involved in CXPA tumorigenesis. Recent progress in tumour microenvironment research has led to identification that extracellular matrix (ECM) remodelling and increased stiffness act as critical contributing role in tumour carcinogenesis. This study examined ECM modifications to elucidate the mechanism underlying CXPA tumorigenesis.

**Results:**

PA and CXPA organoids were successfully established. Histological observation, immunohistochemistry (IHC), and whole-exome sequencing demonstrated that organoids recapitulated phenotypic and molecular characteristics of their parental tumours. RNA-sequencing and bioinformatic analysis of organoids showed that differentially expressed genes are highly enriched in ECM-associated terms, implying that ECM alternations may be involved in carcinogenesis. Microscopical examination for surgical samples revealed that excessive hyalinized tissues were deposited in tumour during CXPA tumorigenesis. Transmission electron microscopy confirmed that these hyalinized tissues were tumour ECM in nature. Subsequently, examination by picrosirius red staining, liquid chromatography with tandem mass spectrometry, and cross-linking analysis indicated that tumour ECM was predominantly composed of type I collagen fibers, with dense collagen alignment and an increased level of collagen cross-linking. IHC revealed the overexpression of COL1A1 protein and collagen-synthesis-related genes, DCN and IGFBP5 (*p* < 0.05). Higher stiffness of CXPA than PA was demonstrated by atomic force microscopy and elastic imaging analysis. We utilized hydrogels to mimic ECM with varying stiffness degrees in vitro. Compared with softer matrices (5Kpa), CXPA cell line and PA primary cells exhibited more proliferative and invasive phenotypes in stiffer matrices (50Kpa, *p* < 0.01). Protein-protein interaction (PPI) analysis of RNA-sequencing data revealed that AR and ERBB-2 expression was associated with TWIST1. Moreover, surgical specimens demonstrated a higher TWIST1 expression in CXPA over PA. After knocking down TWIST1 in CXPA cells, cell proliferation, migration, and invasiveness were significantly inhibited (*p* < 0.01).

**Conclusion:**

Developing CXPA organoids provides a useful model for cancer biology research and drug screening. ECM remodelling, attributed to overproduction of collagen, alternation of collagen alignment, and increased cross-linking, leads to increased ECM stiffness. ECM modification is an important contributor in CXPA tumorigenesis.

**Supplementary Information:**

The online version contains supplementary material available at 10.1186/s13578-023-01071-x.

## Introduction

Pleomorphic adenoma (PA), which frequently harbors recurrent translocations of the *PLAG1* gene on 8q12 (> 50%) [1], is the most common benign tumour among salivary gland tumours, with a prevalence of 60% [2]. About 6.2% of PA may transform into carcinoma ex pleomorphic adenoma (CXPA) [3], which is characterized by aggressive behavior, a poor prognosis, and an 18% 10-year survival rate [4]. Investigations into the molecular mechanisms underlying the malignant transformation of PA to CXPA will help to establish a prognostic assessment model and identify effective therapeutic targets to improve patient survival.

In most cases during PA carcinogenesis, the luminal (or ductal) components of PA undergo malignant transformation and exhibit the histology of salivary duct carcinoma (SDC) or poorly differentiated adenocarcinoma [4]. Some genes have been reported to be abnormally expressed in CXPA rather than PA. Androgen receptor (AR) is overexpressed in CXPA, and its positivity is a useful diagnostic marker for CXPA [5, 6]. Both literature and our previous studies have demonstrated that human epidermal growth factor receptor 2 (HER-2, encoded by *ERBB-2* gene) amplification promotes CXPA carcinogenesis by enhancing cell proliferation and invasiveness via activating the PI3K/Akt and MAPK/ERK signalling pathways [4, 7, 8]. E-cadherin (E-cad), a well-known adhesion molecule, is down-expressed during carcinogenesis due to the epigenetic and/or genetic alterations in the CDH1 gene [9]. Additionally, a positive correlation has been established between the downregulation of E-cad expression and the histological grade and TNM stage of CXPA patients [10]. However, the molecular mechanisms underlying CXPA tumorigenesis remained obscure.

Liotta et al. postulated that the malignant transformation of a tumour reflects the ability of neoplastic cells to overcome growth restrictions in the tumour microenvironment (TME) [11]. Scarini et al. propose that during CXPA carcinogenesis, several molecular signals from TME may induce malignant transformation of benign PA cells [12]. For instance, tenascin, an extracellular matrix (ECM) glycoprotein, was found to be expressed in the stroma of CXPA, which may contribute to neoplastic proliferation [13]. In one of our previous studies, we conducted RNA-sequencing of fresh specimens of CXPA and adjacent normal tissues and found that ECM-related terms, such as ECM organization and collagen fibril organization, were highly enriched in the transcription network of CXPA [14]. These findings implied that disruption of the ECM may be part of the mechanisms driving CXPA tumorigenesis.

The ECM plays a central role in the formation of the TME and provides a supportive environment for cell survival. ECM remodelling caused by cancer and mesenchymal cells may generate a highly fibrotic tumour microenvironment, thereby increasing matrix stiffness [15]. It has been demonstrated that elevated ECM stiffness can induce mechanical stimulation, thereby activating the receptors and mechanosensors on the cell membrane and influencing diverse cellular processes, such as cell proliferation, survival, and invasion [15]. However, it remains unclear whether CXPA carcinogenesis is associated with ECM alternations.

Since our findings [14] regarding ECM in CXPA were based on fresh samples, which may include mesenchymal tissues, thus RNA-sequencing and bioinformatic analysis may produce false positive results. Patient-derived organoids model predominantly consists of tumour cells. Studies on different patient-derived organoids have revealed that they contain cancer stem cells and share the same histological features as their parental tumours [16]. Although the model has been primarily used for drug screening and personalized medicine [17], organoids are now employed for biomarker analysis and investigations into the molecular mechanisms of cancers [18, 19]. Bartfeld et al. used normal organoids from the stomach to investigate the occurrence and progression of gastric cancer [20]. Upper tract urothelial carcinoma organoids combined with scRNA-sequencing analysis revealed that gemcitabine treatment increased the expression of c-Met, thereby inducing drug resistance [21]. Among salivary gland tumours, organoids of mucoepidermoid carcinoma, and adenoid cystic carcinoma (ACC) have been built up [22, 23]. However, no study has reported the establishment of organoids of CXPA.

In this study, we first developed PA and CXPA organoids and explored whether they could recapitulate the phenotypic and molecular characteristics of their parental tumours through morphological observation, immunohistochemistry (IHC), and whole-exome sequencing (WES). Subsequently, we performed RNA-sequencing and bioinformatics analysis to investigate the molecular mechanisms associated with PA carcinogenesis. Finally, the hypothesised mechanism of PA carcinogenesis was verified through histomorphological observation, IHC, mass spectrometry, transmission electron microscopy, collagen cross-linking assay, elastic imaging analysis, atomic force microscopy, and cell biology behavioural assays.

## Materials and methods

### Clinical specimens

Between September and December 2021, surgically resected PA (n = 5) and invasive CXPA (n = 4) tissues were harvested from patients at the Shanghai Ninth People’s Hospital, Jiao Tong University, School of Medicine. Fluorescence In Situ Hybridization (FISH) identified 3/4 positive cases for PLAG1 translocation and 2/4 positive cases for HER-2 amplification among CXPAs. The malignant components of four CXPAs were SDC (Details of clinical information listed in Additional file 1: Table S1). All procedures involving human specimens were approved by the ethics committee of Shanghai Ninth People’s Hospital (SH9H-2022-T184-1). The participants provided written informed consent before enrollment.

### PA and CXPA organoids culture

Tumour tissues were sectioned into 3-mm-thick pieces and washed with Dulbecco’s Modified Eagle Medium (DMEM) (Gibco, USA) for organoid cultures. Tumour fragments were dissociated into single cells in DMEM with collagenase II (Sigma, Aldrich, USA) and DNase I (Applichem, Germany) at 37 ℃ and gentle shaking for 2 h. The cells were counted and resuspended in Matrigel (R&D Systems, USA), plated as Matrigel domes in 24-well culture plates, and maintained at 37 ℃, 5% CO_2_ with media covering the Matrigel dome. The components of the organoid culture medium are illustrated in Additional file 1: Table S2. On day seven, the organoid growth was monitored at two or three days intervals. The culture medium was replaced every three to four days. Organoids were passaged at a 1:5 − 1:6 ratio every two weeks. Organoids were dissociated from Matrigel before freezing in Serum-free Cell Cryopreservation Medium (NCM, China) to cryo-preserve them.

### H&E, IHC staining, and WES analysis of organoids and their parental tumours

To determine whether PA and CXPA organoids retained the histological characteristics of the parental tumours, organoids were fixed with 10% formalin for two hours, followed by agar pre-embedding. H&E and IHC staining was also performed on organoids and their parental tumours. We used anti-Ki67, anti-p63, anti-Cytokeratin 7 (CK7), anti-Cytokeratin 14 (CK14), anti-Smooth muscle actin (SMA), and anti-Calponin (Calp) for IHC labelling. PBS instead of a primary antibody was used as blank control. Details of the experimental procedure and antibody information are provided in Additional file 1. Ki67 staining was evaluated using the labelling index, defined as the percentage of tumour cells which showed nuclear immunoreactivity. For the parental tumour, at least 1000 tumour cells were counted across 10 high-magnification fields. Similarly, at least 300 tumour cells were counted in 10 high-magnification fields for organoids. IHC slides were scored by two independent experienced pathologists.

Genomic DNA of PA and CXPA organoids and parental tumours was extracted using a QIAamp DNA Mini Kit (QIAGEN, Germany) for whole-exome sequencing (WES) detection. Sequencing was performed using the HiSeq PE150 system (Illumina, USA), and exonic variants were analysed using the DRAGEN Bio-IT Platform (Illumina, USA).

### Organoids RNA-sequencing analysis and bioinformatics analysis

The total RNA of PA and CXPA organoids was extracted using the TRIzol reagent according to the manufacturer’s protocol. Illumina HiSeq X Ten platform was used for RNA sequencing. The R package Deseq2 was used to normalize the raw data. |log2(FoldChange)|>1 and p < 0.05 were set as the threshold for differential expression analysis. GO and KEGG pathway enrichment analysis of differentially expressed genes (DEGs) were performed using R software based on the hypergeometric distribution. The protein-protein interaction (PPI) network of DEGs was analysed using the STRING database and visualized via Cytoscape software.

### Transmission electron microscope (TEM) observation in ECM regions of CXPA specimens

Fresh CXPA tissues were sliced to a thickness of 1 mm, fixed with 2.5% glutaraldehyde for two hours, and then postfixed with 1% osmium tetroxide for two hours. The specimen was embedded in Epon resin, subsequently sliced into 80 nm pieces, and finally imaged at 100 kV under a TEM (FEI Talos 120).

### Picrosirius Red (PSR) staining of ECM regions of PA and CXPA specimens

Collagen fibers could be stained red under light microscopy by PSR assay. Under polarized light microscopy, type I collagen appeared red/orange, whereas type III collagen was green. PSR staining was also utilized to evaluate the orientation of collagen fiber alignment. Polarized light microscopy images were converted to 8-bit for the alignment analysis. The angles of collagen fibers (type I) were measured using the angle tool in Image J (version 1.51j, NIH, USA), and all angles were referenced to a horizontal line. Three fields rich in collagen fibers were analyzed per case. In each field, the angles of at least 20 fibers were measured, and the alignment index (AI) was calculated using the following equation:

$$AI=\frac{{\sum }_{i=1}^{N}{\text{cos}\left({\theta }_{i}-{\theta }_{median}\right)}^{2}}{N}$$

N is the number of fibers measured, ${\theta }_{i}$ is the angle of an individual fiber, ${\theta }_{median}$ is the median angle of the image. AI = 0 refers to random orientation and AI = 1 describes fully aligned fibers.

Paraffin-embedded samples of PA and CXPA organoids and their parental tumours were sectioned into 4 μm slices and stained with PSR using a commercial kit (Solarbio, China), following the manufacturer’s instructions.

### Collagen cross-linking analysis

Normally, soluble non-cross-linked collagen can be decomposed by pepsin. Therefore, pepsin could divide total collagen content into soluble (non-cross-linked) and insoluble (cross-linked) components. The insoluble collagen content is calculated by subtracting the soluble collagen content from the total collagen content. The sircol soluble collagen assay kit (Biocolor, S1000, UK) was used to determine the soluble collagen content. The levels of total collagen were determined using a Hydroxyproline assay kit (Nanjing Jiancheng, A030-2-1, China). Studies were conducted on CXPA (n = 5) and PA (n = 5) tissue samples, following the manufacturer’s instructions.

### Identification and validation of the potential genes related to ECM remodelling and TWIST1 in PA and CXPA surgical specimens

IHC was performed on archived paraffin-embedded samples of PA (n = 14) and CXPA (n = 14) to assess the expression of the *COL1A1*, *DCN*, and *IGFBP5*, which are related to ECM remodelling and transcription factor *TWIST1*. The IHC staining was developed according to standard procedures. Supplementary Table S3 provides the details of the primary antibodies mentioned previously. PBS instead of the primary antibody was used as blank control. Two pathologists examined immunohistochemical sections to determine the percentage of positive cells and staining strength. The percentage of positive cells was determined according to previous reports [9]. The percentage score was multiplied by the intensity score and sections were divided into 2 groups based on the resulting product, as follows: low expression (score≥4) and high expression (score ≥ 4).

Liquid Chromatography with tandem mass spectrometry (LC-MS/MS) analysis was used to identify the amounts of *COL1A1*, *DCN*, and *IGFBP5* proteins in CXPA and PA clinical specimens. The details are illustrated in Additional file 1.

### Quantitative assessment of PA and CXPA stiffness with shear-wave elastography (SWE) and atomic force microscopy (AFM)

The radiologists performed SWE testing on one CXPA and one PA patient pre-operatively. The elastographic images were obtained using the Aixplorer system (SuperSonic Imagine, Aix en Provence, France) with a 50 mm 15 − 4 MHz linear-array transducer. The scan was converted to SWE mode, with the probes placed lightly and perpendicularly to the skin surface for scanning and measuring. The patient was required to mute and relax the neck muscles during this time, and the colours in the SWE sampling frame were filled and stable for three seconds. The average value was then calculated after the Q-Box was placed in an area with the highest lump velocity value in the sampling frame and measured three times with a diameter of about 3–5 mm;

The stiffness of the CXPA and PA was also quantified on their paraffin-embedded samples. Briefly, paraffin-sectioned samples were deparaffinized, rehydrated, and dried. AFM measurements were performed in the air by a commercial AFM setup (NT-AIST, HORIBA, Japan) in the force mapping mode using a high-quality tip (MikroMasch, USA). The spring constant of the cantilever was 5 N/m. The elastic modulus (E-modulus) of the tissue sample was calculated from the force-indentation curve using the Hertz model [24],

$$\text{F}\text{=}\frac{\text{4}}{\text{3}}\frac{\text{E}}{\text{1-}{\nu }^{\text{2 }}}\sqrt{\text{R}}{\text{?}}^{\text{3/2}}$$

where the measured force is *F*, the elastic modulus *E*, the sample indentation *δ*, and the radius of the spherical tip is *R*. The Poisson’s ratio for an incompressible sample was set to $\nu$ = 0.5. To assess the E-modulus from one PA and one CXPA sample, ten force maps (each with 50 × 50 force-indentation curves on a scan of 10 × 10 µm 2) were recorded at different locations within the ECM-rich region of each tissue. For each force map an average E-modulus was calculated. The average E-modulus was normalized by dividing by the number of tumour cells on a scanning area of 10 × 10 µm [2].

### Validation of simulated microenvironment stiffening in vitro influencing cell proliferation, migration and invasion of PA primary cells and CXPA cell line

Hydrogels were utilized to simulate a matrix with varying stiffness using a GM90 hydrogel kit (EFL, Suzhou, China) based on the manufacturer’s instructions.

Stiffness measurements of hydrogels were performed by DHR-2 rheometer (TA, USA). GelMA solution (at a final concentration of 20%) supplemented with lithiumphenyl (2,4,6-trimethylbenzoyl) phosphinate was added to the DHR-2 rheometer, and the UV illumination (405 nm) lasted for 3 and 20 s, respectively. During irradiation, the stiffness of hydrogels was measured by a DHR-2 rheometer (Temp = 25 ℃, Strain % = 1.0%, Angular frequency = 5.0 rad/s). Irradiation for 3 s resulted in a hydrogel stiffness value of 5 Kpa; irradiation for 20 s resulted in a value of 50 Kpa.

PA primary cells and CXPA cell line (SM-AP1) were cultured for cell proliferation, migration, and invasion assays. Detailed information about cell culture is provided in Additional file 1. The human CXPA cell line, SM-AP1, was a gift from Niigata University, Japan [25].

To directly observe cell viability, SM-AP1 cells and PA primary cells (1 × 10^5^ cells/well) were seeded on a 12-well plate coated with 20% GM90 hydrogels and were stained using a live/dead assay kit (Yeasen, Shanghai, China) according to the manufacturer’s instructions.

A wound-healing assay was performed to assess cell migration. SM-AP1 cells and PA primary cells were seeded at 3 × 10^5^ cells/well density into the culture inserts of 12-well plates coated with 20% GM90 hydrogels and incubated until 90% confluence. After 24 h, the cells were assessed using live/dead staining, and the relative scratch healing area was analysed using Image J software.

A transwell assay was conducted to determine the invasiveness of cells according to standard procedures. SM-AP1 cells were pre-incubated on 20% GM90 hydrogels for 48 h, and PA primary cells following the same treatment were seeded at a density of 3 × 10^4^ cells/well in 24-well plates. After 24 h of culturing and 15 min of crystal violet staining, images were captured to evaluate the invasion. We divided the number of invaded cells by the proliferation rate^*^ (^*^proliferation rate = *proliferation rate of cells in stiffer matrices/proliferation rate of cells in softer matrices*) to normalize invasion results.

### Evaluation of effects of cell proliferation, migration and invasion after knocking down TWIST1 in CXPA cell line

TWIST1 knockdown was mediated by siRNA transfection. Three siRNA sequences were designed to silence TWIST1 mRNA:

TWIST1-si-1(5’-3’): GCAAGAUUCAGACCCUCAATT; UUGAGGGUCUGAAUCUUGCTC.

TWIST1-si-2(5’-3’): GGAGUCCGCAGUCUUACGATT; UCGUAAGACUGCGGACUCCCG.

TWIST1-si-3(5’-3’): UCCAAGAUGGCAAGCUGCAGCUAUGTT; CAUAGCUGCAGCUUGCCAUCUUGGAGT.

NC(5’-3’): UUCUCCGAACGUGUCACGUdTdT.

SM-AP1 cells were seeded in 6-well plates, and at 40% confluency, they were transfected with targeted siRNA and non-targeted siRNA (NC) for 48 h, respectively. The efficiency was evaluated by real-time PCR.

Cell counting kit 8 (CCK8) was applied to determine the impact on cell proliferation. Following the manufacturer’s instruction (CCK-8; NCM, Suzhou, China), both SM-AP1 cells (NC) and cells transfected with siTWIST1 were seeded in 96-well plates (1 × 10^4^ cells/well), and 10 µl of CCK-8 reagent was added to each well. After a 2 h incubation, the absorbance was detected spectrophotometrically at 450 nm (SpectraMax i3, Molecular Devices, USA). The cells were harvested for CCK8 assay after culturing for 0, 24, 48, and 72 h, respectively.

Transwell assay of SM-AP1 cells transfected with or without siTWIST1 was performed according to standard protocol. Furthermore, the ability of cell migration and invasion was assessed after 24 h of culturing at 37 °C and 15 min of crystal violet staining, respectively. We divided the number of invaded cells by the proliferation rate^*^ (^*^proliferation rate = *proliferation rate of NC cells/proliferation rate of TWIST-knockdown SM-AP1 cells*) to normalize invasion results.

### Statistical analysis

Statistical analyses were performed with the R (v4.1.2) and Prism 9.0 (GraphPad, San Diego, CA) softwares. Unless otherwise specified, comparisons were conducted using the Student’s t-test and an unpaired two-sided Wilcoxon rank-sum test. ^*^p < 0.05, ^**^p < 0.01, ^***^p < 0.001, and ^****^p < 0.0001 indicate statistical significance in the figures.

## Results

### Establishment of patient-derived PA and CXPA organoids

**Fig. 1:**
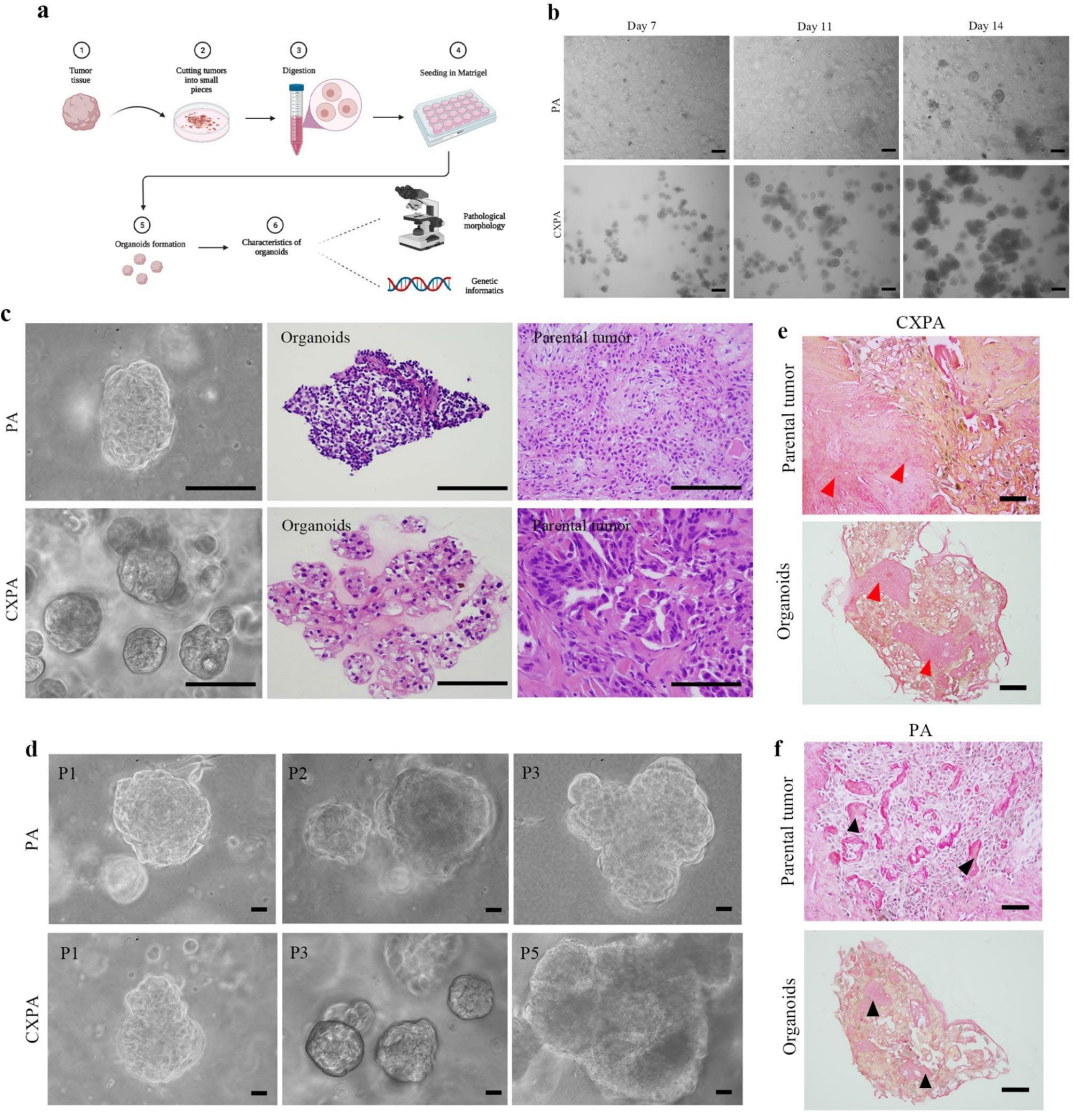
Establishment of patient-derived PA and CXPA organoids. **(a)** Schematic overview of the tumour-derived organoid culture. **(b)** Bright-field images of PA and CXPA organoids in the first generation on days 7, 11, and 14. Scale bars = 100 *µ*m. **(c)** Overview of PA and CXPA organoids under bright-field and H&E staining results of PA and CXPA organoids and their parental tumours. Scale bars = 100 *µ*m. **(d)** Bright-field images of PA (generations 1, 2, and 3) and CXPA (generations 1, 3, and 5) organoids. **(e)** Representative image of PSR-stained positive areas (in red colour, red arrowhead) of CXPA organoids and their parental tumours. **(f)** Representative image of PSR-stained positive areas (in red colour, black arrowhead) of PA organoids and their parental tumours. Scale bars = 100 *µ*m

The schematic overview of organoids establishment and identification is depicted in Fig. 1a. Individual tumour cells encapsulated in Matrigel began to proliferate and divide on days 1 to 3 of organoids culture, eventually forming cell clusters. Subsequently, the cell masses continued to grow in size. On day 14, the organoids continued to proliferate (Fig. 1b). Under a light microscope, PA and CXPA organoids exhibited a solid and nodular appearance (Fig. 1c). The first passage of organoids was performed after two weeks of culture with a split ratio of 1:6. To date, human CXPA tumour-derived organoids have been successfully passaged four times, and PA organoids were passaged to the third generation (Fig. 1d). The fifth passage of CXPA organoids as well as the third generation of PA organoids, did not maintain sustained proliferative activity.

### PA and CXPA organoids preserve histological characteristics of parental tumours

In contrast to PA organoids, CXPA organoids displayed a larger cell volume and nucleus under the microscope, indicating greater cellular atypia. Conversely, PA organoids showed uniform cell morphology (Fig. 1c). Hyaline material deposits in certain areas of the CXPA and PA organoids between tumour cells. The hyaline material was stained red by PSR, and PSR positivity was detected not only in PA and CXPA organoids but also in their parental tumours (Fig. 1e, f).

**Fig. 2:**
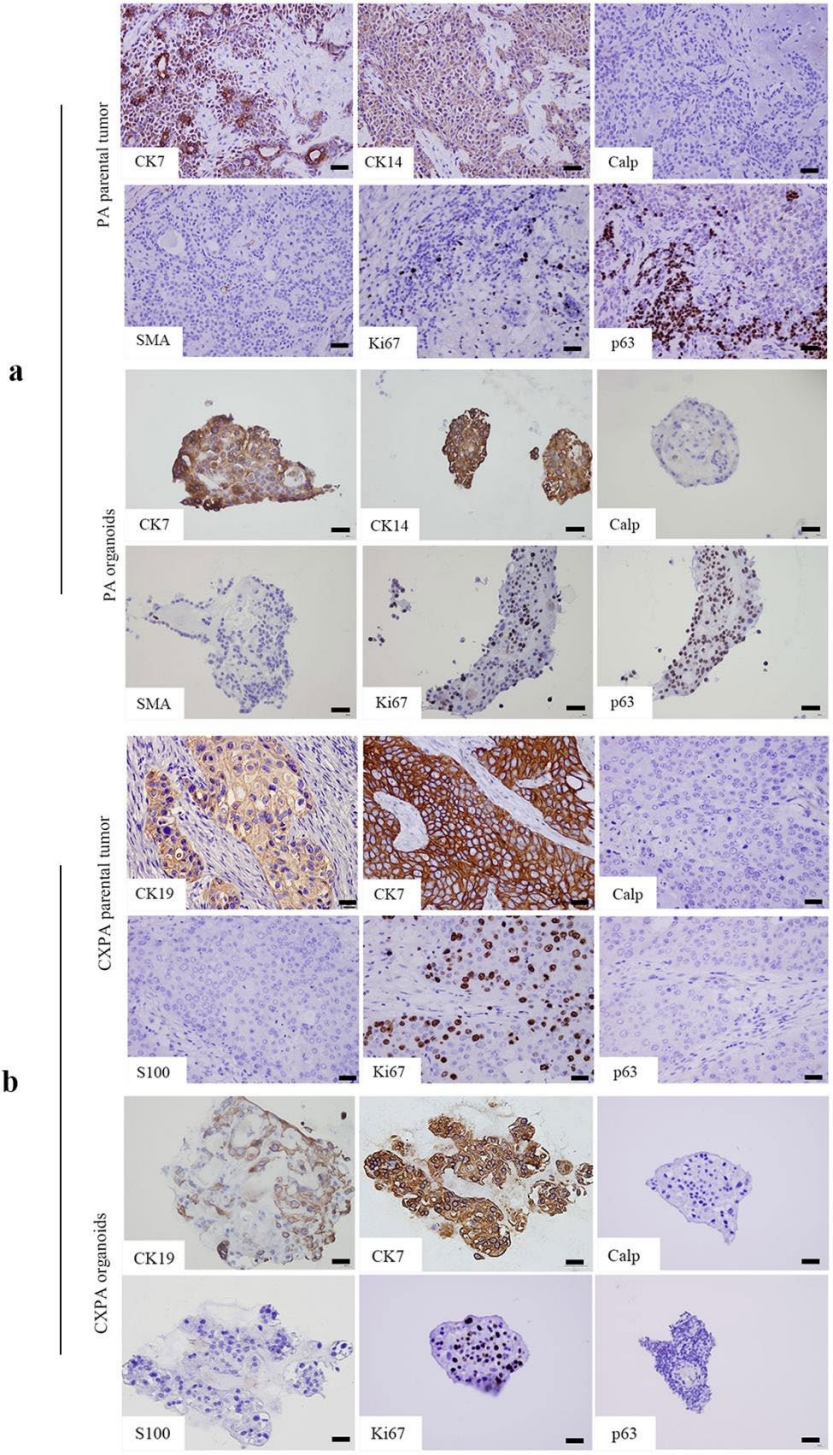
Patient-derived PA and CXPA organoids preserved the histopathological characteristics of their parental tumours. **(a)** CK7, CK14, Ki67, and p63 were positive in PA organoids and their parental tumours, while Calp and SMA were negative. **(b)** CK19, CK7, and Ki67 were positive in CXPA organoids and their parental tumours, while p63, S100 and Calp were negative. Scale bars = 100 *µ*m

Normally, PA typically expresses CK7, CK14, SMA, p63, and Calp at various degree. However, SDC of CXPA expresses CK7 and CK19 instead of S100, p63, and Calp. Figure 2a depicts a case of PA in which CK7, CK14, and p63 were positive while SMA and Calp were negative in both organoids and their parental tumour. On the other hand, CXPA organoids only express CK7 and CK19, similar to their parental tumours (Fig. 2b). The Ki67 proliferative index was higher in CXPA organoids (Mean ± SD, 36.67% ± 0.02) than in PA organoids (Mean ± SD, 7.33% ± 0.01, *p < 0.01*), indicating higher growth activity of malignant cells. The morphology and IHC analysis revealed that both PA and CXPA organoids retained the phenotypic features of their parental tumours.

### PA and CXPA organoids retain the mutational spectra of their parental tumours

**Fig. 3:**
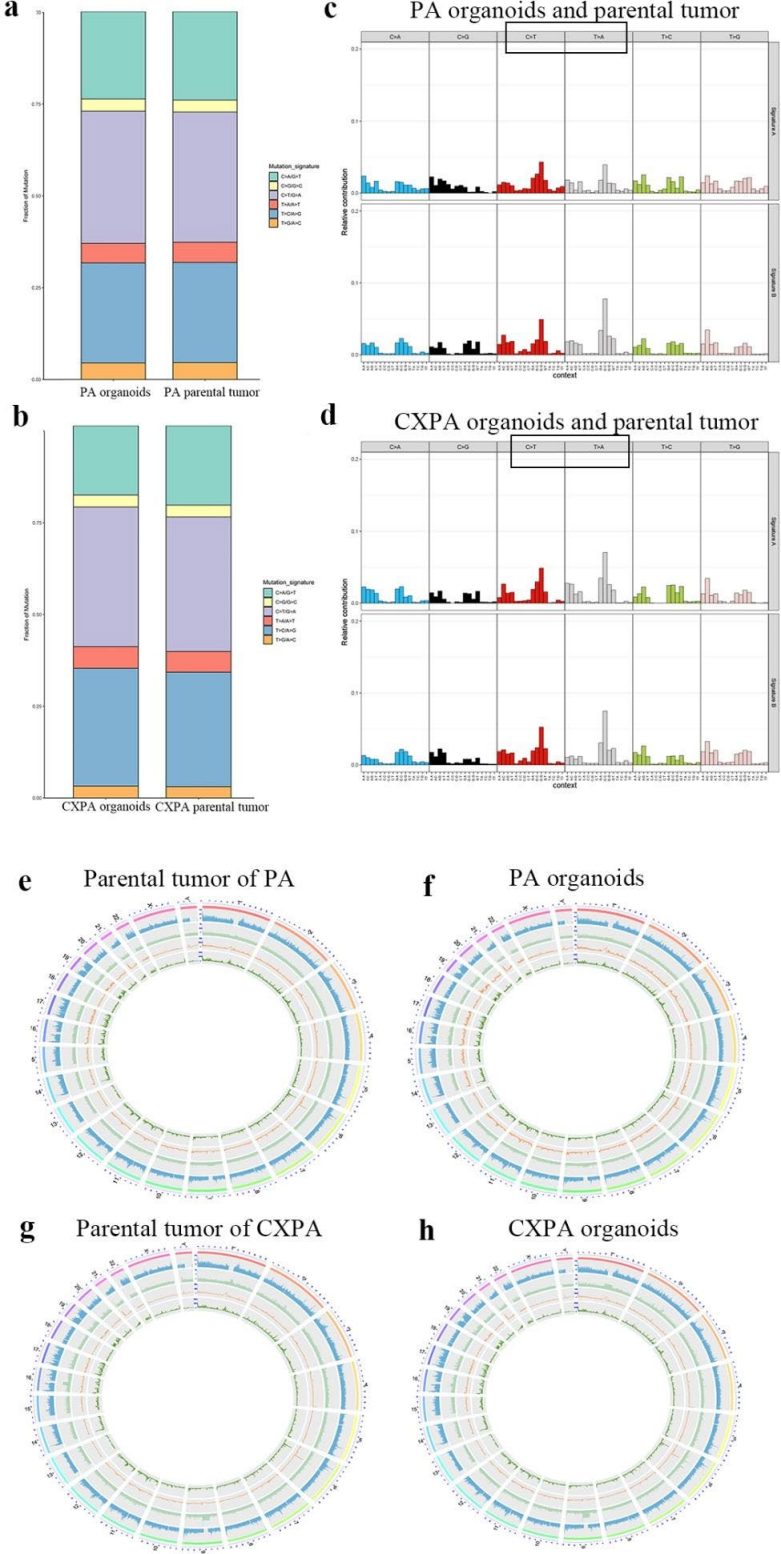
PA and CXPA organoids exhibited accurate recapitulations of genetic alterations of their parental tumours. **(a)** Mutation spectrum analysis of primary tumour (left column) and organoids (right column) of PA. **(b)** Mutation spectrum analysis of parental tumour (left column) organoids (right column) of CXPA. **(c)** Characteristics of 96 trinucleotide mutations in PA organoids and parental tumour. **(d)** Characteristics of 96 trinucleotide mutations in CXPA organoids and parental tumour. The most common mutation types in **(c)** and **(d)** are shown in a black frame. e-h. The Circos plot is divided into five layers from outside to inside, illustrating the chromosome bands (the chromosome number and chromosome-scale), sequencing coverage, indel distribution (the value is the number of indel within 500 kb bases), SNP distribution (the number of SNP within 500 kb bases) and CNV distribution (the number of CNV within 500 kb bases. **(e)** Parental tumour of PA, **(f)** PA organoids, **(g)** Parental tumour of CXPA, and **(h)** CXPA organoids

We performed WES on organoids, parental tumours, and para-cancerous tissues. Additionally, mutation spectrum, mutation signatures, and copy number variations (CNV) on somatic mutations were analysed to determine if organoids represented the genome spectrum of their parental tumours. The mutation spectrum of six base substitutions was characterized in all tumour samples. Consequently, PA and CXPA organoids were comparable to their parental tumours regarding point mutations. C > T/G > A and T > C/A > G transitions were the most common mutation types in PA organoids and their parental tumours, while the C > G/G > C transversion was the least prevalent (Fig. 3a). Similar mutation types were also observed in CXPA organoids and their parental tumours (Fig. 3b).

Subsequently, point mutations were clustered into multiple different mutation features based on the frequency of 96 mutation types in each tumour sample using the non-negative Matrix Factorization (NMF) method. Consequently, the distribution of mutation signatures of PA and CXPA organoids was highly synchronised with that of their parental tumours. Signatures A and B were extracted from PA organoids and their parental tumours (Fig. 3c) and from CXPA organoids and their parental tumours (Fig. 3d). Signatures A and B were characterized by high rates of C > T transition and T > A transversion. Signatures A and B were similar to COSMIC mutational signature 5. Subsequently, the CNV information was integrated into Circos maps to display genomic data (Fig. 3e h), and it was found that CNV distribution in the PA and CXPA organoids was consistent with their parental tumours, respectively.

### Identification of DEGs and functional and pathway enrichment analysis of PA and CXPA organoids

**Fig. 4:**
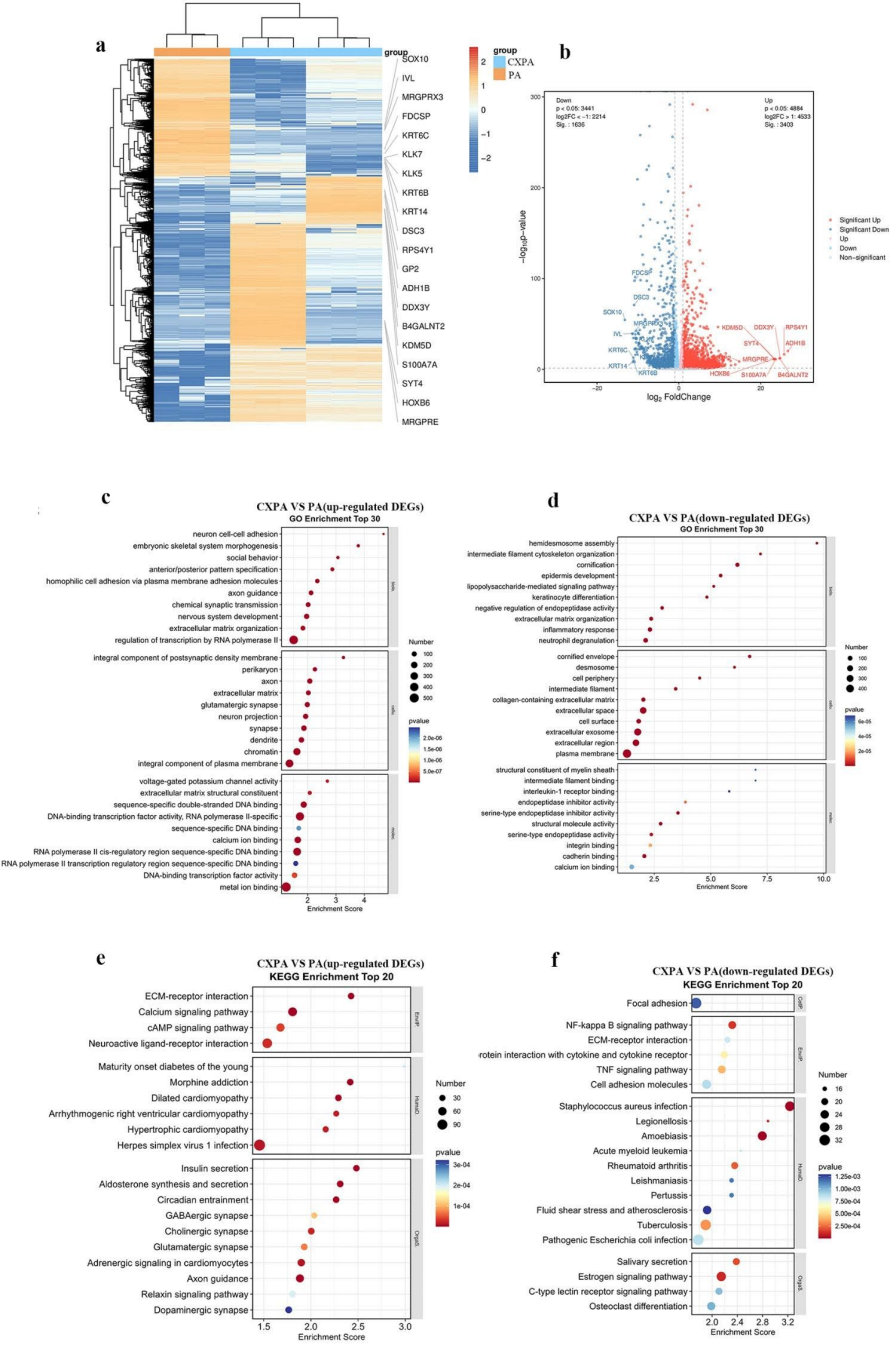
Heatmap and volcano plot of DEGs in PA and CXPA organoids and the top-ranked GO terms and KEGG pathways. **(a)** The heatmap (yellow, up-regulated; blue, downregulated). **(b)** The volcano plot (p-value < 0.05, |log2FC|>1; red and blue squares). The p-value was adjusted using the Benjamini-Hochberg method. Bubble plots displaying the top 30 GO terms in 3403 up-regulated DEGs **(c)** and 1636 downregulated DEGs **(d)**. Bubble plots displaying the top 20 enriched KEGG pathways in 3403 up-regulated DEGs **(e)** and 1636 downregulated DEGs **(f)**

We performed RNA sequencing on PA and CXPA organoids. A total of 5039 DEGs between CXPA and PA organoids were identified, including 3403 up-regulated and 1636 downregulated genes (Fig. 4a). The top 10 up-regulated and downregulated genes are shown in volcano plots (Fig. 4b). The potential functional and molecular pathways associated with DEGs were validated using GO and KEGG pathway enrichment analyses. GO enrichment analysis revealed that up-regulated DEGs were enriched in the regulation of transcription by RNA polymerase II, extracellular matrix organization, extracellular matrix, extracellular matrix structural constituent, and metal ion binding (Fig. 4c). In contrast, downregulated DEGs were primarily involved in the extracellular matrix organization, extracellular space, extracellular region, plasma membrane, and integrin binding (Fig. 4d). KEGG pathway analysis revealed that up-regulated DEGs were enriched in ECM-receptor interaction, calcium signalling pathway, Herpes simplex virus 1 infection, axon guidance and relaxin signalling pathway (Fig. 4e). In contrast, downregulated DEGs were predominantly enriched in focal adhesion, cell adhesion molecules, and ECM-receptor interaction (Fig. 4f).

The GO analysis indicated that up-regulated and downregulated DEGs have a tendency to be involved in ECM-associated terms, such as extracellular matrix organization, extracellular matrix structural constituent, and extracellular space. The KEGG analysis of up-regulated DEGs showed enrichment in ECM-receptor interactions. These results suggested that ECM remodelling may be important in PA carcinogenesis.

### Variations of ECM act as a key event involved in CXPA tumorigenesis

**Fig. 5:**
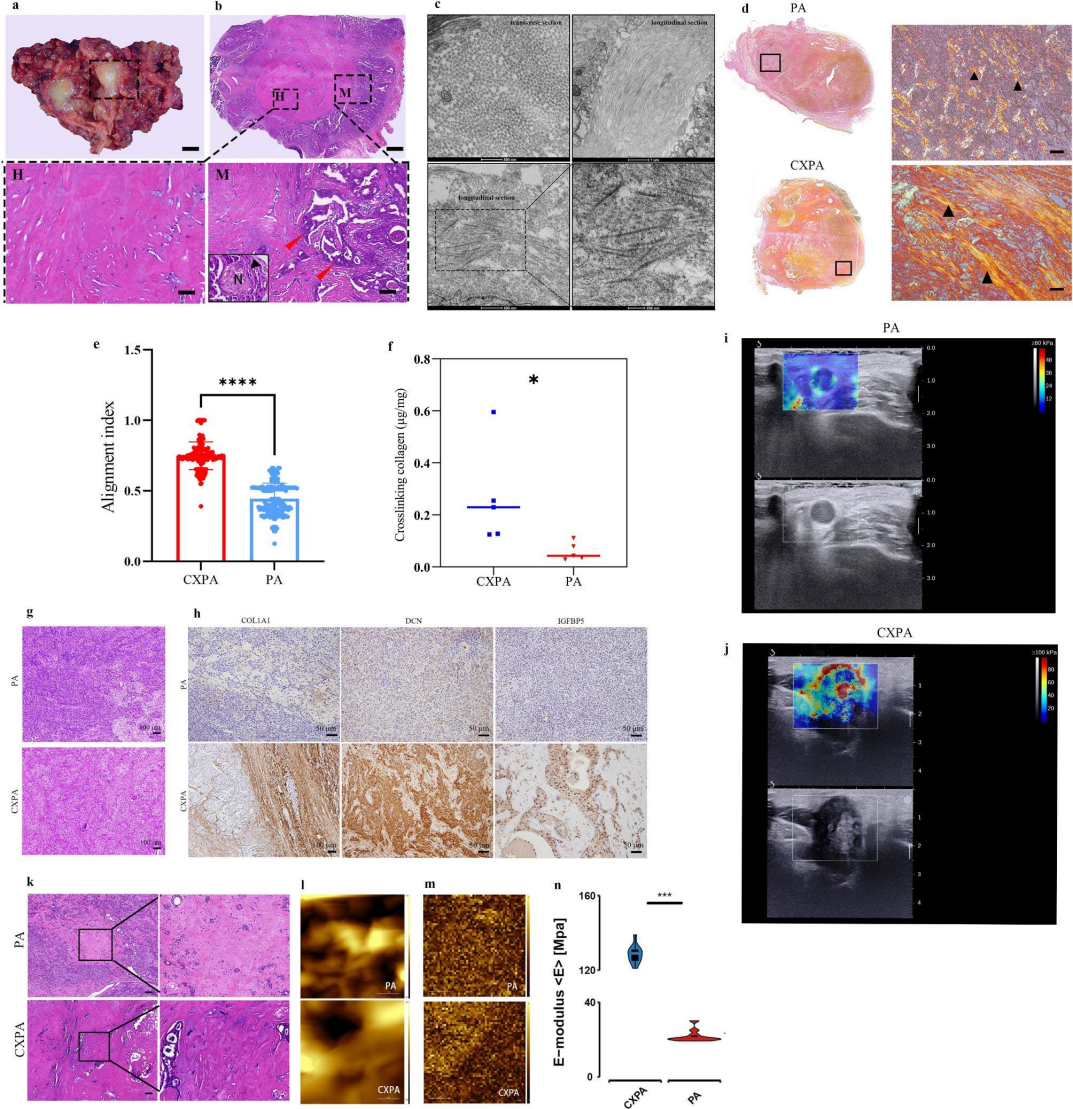
ECM alterations in CXPA tumorigenesis and stiffness measurements of PA and CXPA. (a) Macroscopic appearance of surgically resected CXPA with SDC tissue specimen. Scale bars = 5 mm. (b) H&E staining results of CXPA tissue in the black frame in Figure **(a)** Scale bars = 1000 *µ*m. Hyaline changes are shown in (H) of **(b)** Scale bars = 100 *µ*m. A malignant area is shown in (M) of b. Scale bars = 20 *µ*m. Red arrowheads indicate carcinoma extending beyond the pleomorphic adenoma capsule. The image of perineural invasion as an insert section in (M) of b. N represents the nerve, and the black arrowhead indicates the presence of invasive cancer cells. **(c)** TEM images of hyalinized area in CXPA. (Left) collagen in TEM transverse section. Scale bars = 100 nm; (right) collagen in TEM longitudinal section, the collagens have a typical near-circular profile. Scale bars = 250 nm. **(d)** Overview of PSR staining of CXPA and PA specimens. Positive regions under a polarized light microscope were in bright orange (black arrowhead). Scale bars = 50 *µ*m. **(e)** Alignment index (AI) confirmed a higher degree of collagen fiber alignment in CXPA compared to PA tissues (n = 3, respectively). **(f)** Scatter plot of collagen cross-links of PA (n = 5) and CXPA (n = 5) specimens. **(g)** H&E staining of PA and CXPA clinical samples. Scale bars = 100 *µ*m. **(h)** Protein expression of COL1A1, IGFBP5, and DCN in CXPA and PA clinical specimens. Scale bars = 50 *µ*m. **(i)** Shear wave elastography (SWE; top) and B-mode (bottom) images on split screen mode of PA. **(j)** Shear wave elastography (SWE; top) and B-mode (bottom) images on split screen mode of CXPA. k. H&E staining of ECM-rich regions in PA and CXPA specimens. Scale bars = 200 *µ*m. l. AFM topography images of PA (top) and CXPA (bottom). Scale bars = 2 *µ*m. m. Maps of E-moduli of PA (top) and CXPA (bottom). Scale bars = 2 *µ*m. n. Mean E-modulus of ECM-rich regions of PA and CXPA. ^*^p < 0.05, ^***^p < 0.001, ^****^p < 0.0001. Student’s t-test. Error bars indicate mean ± SD.

Histopathologically, under gross examination, the previous PA component was visible and appeared as a sclerotic, scar-like nodule (Fig. 5a) at the cut surface of CXPA. Microscopically, the previous tumour of PA regressed and was replaced by abundant hyalinized tissues (Fig. 5b, marked with H), resulting in a sclerotic appearance under the naked eye. The hyalinized tissues contained strands, nests, or tubules of tumour epithelium, which showed evident atypia (Fig. 5b, marked with M). This tumour epithelium was composed of carcinomatous cells derived from the malignant transformation of PA epithelium. The malignant cells had an invasive growth pattern, permeating the residual PA and infiltrating adjacent normal tissues. Perineural invasion was found in CXPA cases (Fig. 5b, labelled with N). Histologically speaking, the excessive formation of hyalinized tissues in CXPA was the hallmark of CXPA tumorigenesis.

Under TEM observation, the hyalinized tissues of CXPA were predominantly composed of collagen fibres with parallel or crisscross patterns (Fig. 5c), confirming that the hyalinized tissues were tumour ECM. By PSR staining, the ECM collagen appeared bright orange under a polarized light microscope, indicating that type I collagen comprises the majority of collagen (Fig. 5d). Further quantification of the PSR-stained tissue sections revealed a significant change in the collagen fiber orientation in CXPA. There was a significant increase in the number of aligned collagen fibers (AI, Mean ± SD, 0.748 ± 0.098) when comparing to randomly oriented fibers (AI, Mean ± SD, 0.445 ± 0.110) in PA tissues (*p* < 0.01, Fig. 5e) Through collagen cross-linking analysis, a higher amount of cross-linking (insoluble) collagen we observed in the CXPA clinical specimens compared to PA clinical specimens (Fig. 5f), indicating increased collagen cross-linking in CXPA. Collectively, overproduction of type I collagen, alternation of collagen alignment, and an increased level of collagen cross-linking may constitute the major processes that lead to ECM remodelling in CXPA.

We selected DEGs implicated in synthesis of collagen, collagen type I alpha 1 chain (*COL1A1*, FoldChange = 69.1 and *p* < 0.01), and decorin (*DCN*, FoldChange = 135.7 and *p* < 0.01). Type I collagen is a major structural component of the desmoplastic ECM. Additionally, collagen type I is closely related to cell growth, proliferation, and differentiation. DCN regulates collagen fibrillogenesis acting on collagen fibrils diameter and fibrils orientation to achieve the proper assembly of its network. We selected the Insulin-Like Growth Factor Binding Protein 5 (*IGFBP5*, FoldChange = 24.8 and *p* < 0.01), which is a biomarker of pulmonary fibrosis and is also involved in stimulating the growth of intestinal smooth muscle and collagen synthesis. Based on the results of IHC, COL1A1 was positively stained in the region of hyalinized tissues in CXPA. IGFBP5 and DCN were significantly overexpressed in CXPA specimens than in PAs (Fig. 5g, h; Table 1). LC-MS/MS analysis confirmed that COL1A1, DCN, and IGFBP5 were overexpressed in CXPA compared to PA (Table 2).

**Table 1:** COL1A1, DCN, IGFBP5 and TWIST1 detection by IHC for PA (n = 14) and CXPA (n = 14) clinical specimens and correlationship analysis between TWIST1 and AR expression/HER-2 amplification

	PA (No.)		CXPA (No.)	*P* value
	Low high		Low/(-) high/(+)	
COL1A1(IHC)	10 4		3 11	*0.021*
IGFBP5(IHC)	8 6		1 13	*0.013*
DCN(IHC)	10 4		1 13	*0.001*
TWIST1(IHC)	11 3		2 12	*0.002*
AR(IHC) HER-2(FISH)			2 12 3 11	1^#^ *0.002* ^*##*^

*#, correlationship analysis between twist1 and AR expression; ##, correlationship analysis between twist1 and HER-2 amplification*

**Table 2:** LC-MS/MS analysis for DCN, IGFBP5 and COL1A1 proteins

Protein		Gene name	Intensity CXPA	Intensity PA
P24593	IGFBP5		86,891,000	
P02452	COL1A1		160,500,000,000	81,707,000,000
P07585	DCN		51,091,000,000	4,065,100,000

### ECM remodelling results in increased ECM stiffening of CXPA

A B-mode image of PA revealed a regular, well-defined, and hypoechoic mass through SWE detection. SWE depicted a heterogeneous mass with mean and maximum SWE stiffness values of 10.2 and 30.8 kPa, respectively (Fig. 5i). Meanwhile, a B-mode image of CXPA demonstrated an irregular mass, poorly defined, and hypoechoic. SWE depicted a heterogeneous mass with mean and maximum SWE stiffness values of 47.2 and 208.1 kPa, respectively (Fig. 5j). Through AFM detection, force maps of the ECM-rich area of CXPA had different elastic properties compared to the ECM-rich regions of PA (Fig. 5k m). The mean E-modulus of the ECM-rich region of CXPA was significantly greater than that in ECM-rich areas of PA (*p* < 0.001, Fig. 5n). These examinations revealed that the stiffness of CXPA was significantly greater than that of PA.

### Increased stiffness promotes the proliferation, migration, and invasion of PA primary cells and CXPA cell line in vitro

**Fig. 6:**
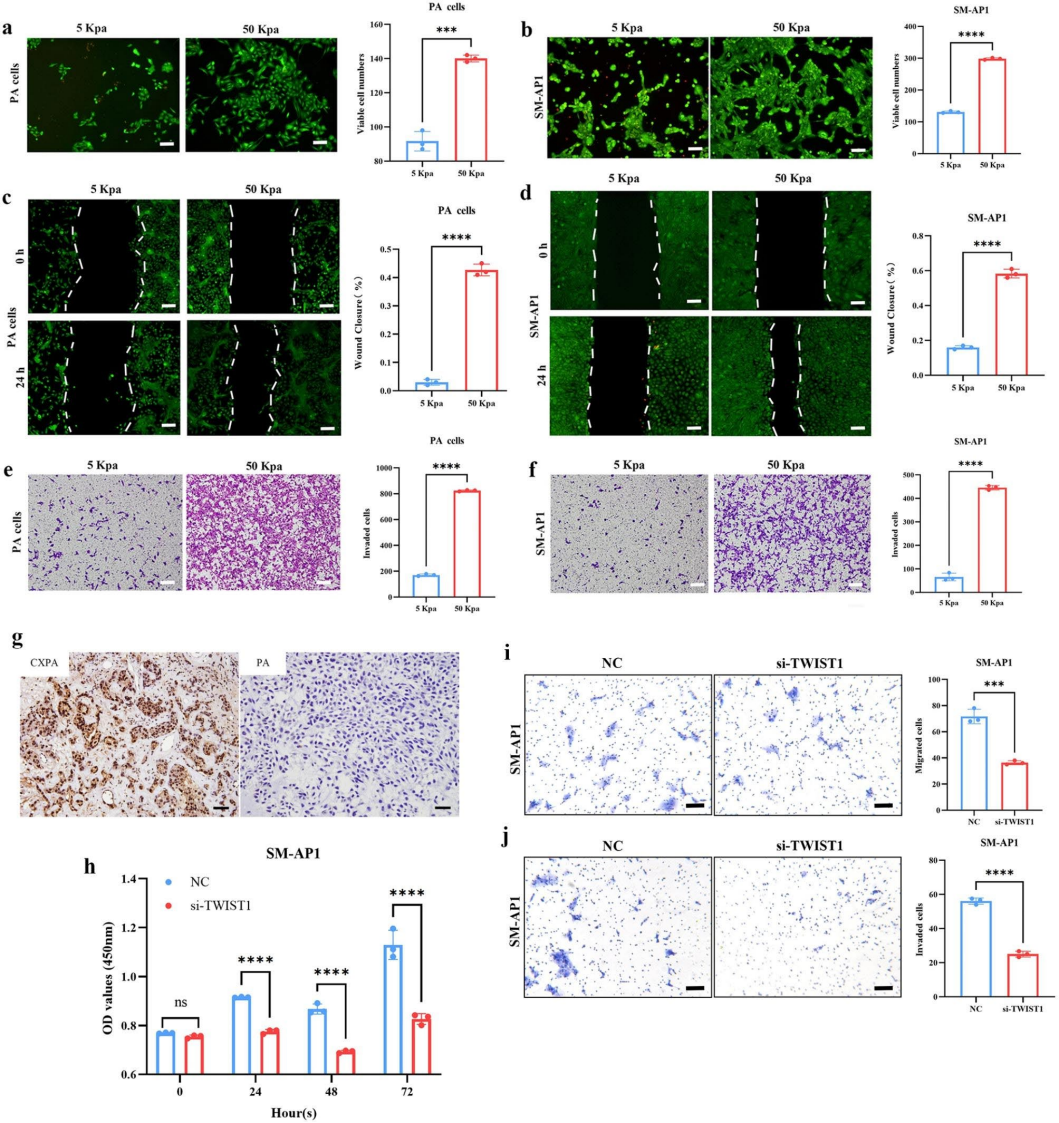
Matrix stiffness and TWIST1 upregulation impacts the biological behaviors of PA primary cells and/or SM-AP1 cells. **(a)** Representative live/dead images of PA primary cells seeded on the surface of GM90 hydrogel. **(b)** Representative live/dead images of SM-AP1 cells seeded on the surface of GM90 hydrogel. **(c)** Representative live/dead images of PA primary cells in 2D scratch assay. **(d)** Representative live/dead images of SM-AP1 cells in 2D scratch assay. **(e)** Invasion of PA primary cells seeded on the surface of GM90 hydrogel. **(f)** Invasion of SM-AP1 cells seeded on the surface of GM90 hydrogel. Scale bars = 50 *µ*m. **(g)** TWIST1 protein staining was higher in CXPA than in PA. Scale bars = 20 *µ*m. **(h)** CCK-8 assay showed that proliferation was reduced in SM-AP1 cells after being transfected with siTWIST1. **(i)** Migration of SM-AP1 with or without knocking down TWIST1 after 24 h. **(j)** Invasion of TWIST1-knockdown SM-AP1 cells and SM-AP1 cells after 24 h. Scale bars = 20 *µ*m. The above independent experiments were repeated three times. ^**^p < 0.01, ^***^p < 0.001, ^****^p < 0.0001, Student’s t-test. Error bars indicate mean ± SD.

After increasing the stiffness of the hydrogels, the number of viable SM-AP1 and PA primary cells increased as determined by the Live/Dead staining assay (Fig. 6a, b). Additionally, the number of migrated cells was higher than in the control group (Fig. 6c, d), as revealed by the wound healing assays. Most SM-AP1 cells and PA primary cells migrated through the membrane in the group with a higher stiffness than the control groups (Fig. 6e, f). These results indicated increased stiffness promoted cell proliferation, migration, and the invasiveness of SM-AP1 and PA primary cells.

### TWIST1 upregulation during CXPA tumorigenesis and relationship with expression of AR and HER-2

Our RNA-sequencing results on organoids revealed that TWIST1 was up-regulated in CXPAs compared to PAs (Fold Change = 102.3, *p* < 0.01). Interestingly, the PPI network analysis of the top 100 genes co-expressed with up-regulated TWIST1 demonstrated AR and ERBB2 gene involvement in this network (Additional file 1: Fig. S1a), implying that TWIST1, AR, and HER-2 may play synergistic roles in PA carcinogenesis. Subsequently, IHC staining of archived samples confirmed that CXPAs express TWIST1 at a higher level than PA specimens (Fig. 6g; Table 1). Our detection on CXPA clinical samples (n = 14, Table 1) also showed that HER-2 amplification was associated with TWIST1 expression (*p* < 0.05), but the relationship between AR expression and TWIST1 was not found (*p* > 0.05). KEGG enrichment analysis performed for the top 100 co-expressed genes with TWIST1 showed the co-expressed genes were involved in terms of pathways in cancer, focal adhesion, and Wnt signalling pathway (Additional file 1: Fig. S1b).

According to the results of real-time PCR (Additional file 1: Fig. S2), we chose TWIST1-si-1 to silence TWIST1 in SM-AP1 cells for subsequent functional experiments. After knocking down TWIST1 in SM-AP1 cells, the CCK-8 assay revealed a reduced growth rate (Fig. 6h). Transwell assays revealed that fewer cancer cells migrated through the membrane in the TWIST1-knockdown group than in control (Fig. 6i, j). These results suggested that TWIST1 downregulation inhibits the proliferation, migration and invasion of the CXPA cell line.

## Discussion

Recent advances in culture technologies have promoted the development of organoid models. Patient-derived tumour organoids benefit from retaining key characteristics of their parental tumours, allowing them to be used in various settings. We established PA and CXPA organoids, which exhibited some genetic and phenotypic similarity to their respective parental tumours. To the best of our knowledge, this is the first study to develop CXPA organoids.

CXPA develops from the malignant transformation of primary or recurrent PA [12]. The mechanisms involved in malignant transformation have not been conclusively established. The tumour microenvironment significantly impacts the emergence and development of malignant tumours [26]. As one of the primary components of the tumour microenvironment, the ECM plays an important role in all biological processes [27]. Histologically, the formation of abundant hyalinized tissues is a distinguishing feature of CXPA. Further investigations confirmed that these hyalinized tissues are the remodelled ECM during carcinogenesis. RNA-sequencing showed that DEGs between CXPA and PA were enriched in ECM-associated genes, which may be associated with successive secretion and re-organization of ECM. Therefore, we suspected that ECM remodelling is a notable characteristic of CXPA carcinogenesis.

Collagen, one of the main ECM components, maintains tissue stability and integrity [28]. Changes in collagen may affect the three-dimensional spatial topology of the matrix surrounding cells, which may influence cell fate [29]. Excess collagen and cross-linking may stiffen the ECM, with the latter being of larger significance [30]. The rigidity of the matrix and its physical stimuli have the same profound effect on cells as chemical signals, and the mechanical microenvironment is associated with tumorigenesis. Provenzano et al. reported that murine mammary epithelial cells in denser and stiffer matrices exhibited more invasive phenotypes [31]. When these cells were subjected to increasing stiffness, the normal epithelial morphology was disrupted, polarization was lost, and matrix invasion occurred [32]. In this study, the overproduction of type I collagen, the alternation of collagen alignment, and the increased level of collagen cross-linking constitute the major processes that lead to ECM remodelling in CXPA. Collagen remodelling may produce a highly fibrotic tumour microenvironment, thereby increasing matrix rigidity. Subsequently, CXPA was confirmed to be significantly stiffer than PA. The CXPA cell line and PA primary cells exhibited more proliferative and invasive phenotypes when incubated on stiffer matrices in vitro. Thus, we presumed that an increase in ECM stiffness mediated by ECM remodelling was closely linked to CXPA carcinogenesis.

As a collagen family member, COL1A1 is closely associated with malignant tumorigenesis [33]. Excessive COL1A1 accumulation in the ECM can lead to various diseases, including cancer [34]. It has been reported that COL1A1 activates the ERK [35] and PI3K/AKT [36] signalling pathways to promote cell transfer and inhibit cell apoptosis. Physiologically, DCN is a member of the small leucine-rich proteoglycan family of proteins and functions as one of the core components of collagen fibril assembly [37]. IGFBP5, a conserved member of the IGFBP family of proteins, promotes fibrosis by enhancing the expressions of ECM genes and pro-fibrotic growth factors, along with the levels of the matrix cross-linking enzyme, lysyl oxidase (LOX) [38]. In this study, the upregulations of COL1A1, DCN, and IGFBP5, as IHC and LC-MS/MS analysis revealed, provided additional evidence that ECM remodelling and stiffness may be involved in CXPA tumorigenesis.

Longevity is an important risk factor for PA malignancy [12]. The incidence of malignancy in PA has been determined to be 1.6% in patients with 5-year tumour durations and 9.6% in patients with > 15 years tumour duration [39]. Neoplastic myoepithelial cells in PA are capable of secreting ECM to form the hyalinized stroma of PA and suppress tumour carcinogenesis. However, divergent signals can alter the suppressor roles of myoepithelial cells, causing them to succumb to autophagy and disappear [12]. Therefore, we postulated that if PA persists, the remodelled and stiffer ECM resulting from continuous deposition of the ECM may act as a signal for disrupting the myoepithelial cell barrier. This prolonged and persistent deposition may be why malignant transformation incidences of PA increase with tumour duration.

AR expression and HER-2 amplification are common in CXPA with SDC component. In this study, the PPI network showed that TWIST1 was co-expressed with AR and ERBB2, and the high expression of TWIST1 was found to be associated with HER-2 amplification, suggesting that TWIST1 may be a pivotal regulator of PA malignancy. TWIST1-knockdown inhibited proliferation, migration, and invasion in the CXPA cell line. TWIST1 is a highly conserved transcriptional factor containing the basic helix-loop-helix (bHLH) domain. It can serve as a sensor and integrator of multiple stimuli and factors from the local microenvironment [40], regulating cell differentiation, migration and proliferation. Activated TWIST1 can transcriptionally regulate various downstream target genes. One of the most common downstream targets of TWIST1 is E-cad, which is the crucial protein in EMT [41, 42]. TWIST1 has a high affinity for the promoter of E-cad, leading to E-cad downregulation. On the other hand, some signals, including Wnt/β-catenin, Notch, and NF-κB, regulate TWIST1 expressions [43]. Our previous studies showed up-regulated FZD2^14^ and down-regulated E-cad [10] in some cases of CXPA. The FZD2 can strongly bind Wnt proteins. Thus, we postulated that TWIST1 might cooperate with FZD2 via the Wnt signalling pathway to down-regulate E-cad. TWIST1 is a potential treatment target for CXPA patients; however, the network signalling pathways mediated by TWIST1 should be further elucidated.

## Conclusion

Our PA and CXPA organoids maintained the characteristics and genetic features of the parental tumours, making them useful tools for the analysis of cancer biology and drug screening. ECM remodelling, attributed to the overproduction of type I collagen, denser arrangement, and increased cross-linking levels of collagen, leads to increased ECM stiffness. The increased ECM stiffness becomes an important mechanism for CXPA tumorigenesis via promoting cell proliferation, migration, and invasion. Our results also indicate TWIST1 acts as a crucial role in carcinogenesis.

## Electronic supplementary material

Below is the link to the electronic supplementary material.

**Additional file 1**: Supplementary methods, tables and figures.

## Data Availability

The data that support the findings of this study are available from the corresponding author upon reasonable request.

## Abbreviations

PA: Pleomorphic adenoma
CXPA: Carcinoma ex pleomorphic adenoma
RNA-seq: RNA sequencing
IHC: Immunohistochemistry
DEGs: Differentially expressed genes
PPI: Protein-protein interaction
STRING: Retrieval of interacting genes/proteins
AR: Androgen receptor
HER2: Human epidermal growth factor receptor 2
H&E: Hematoxylin and eosin
FISH: In situ fluorescence hybridization assay
DC: Salivary duct carcinoma
CNV: Copy number variations
GO: Gene ontology
ECM: Extracellular matrix
SWE: Shear-wave elastography
AFM: Atomic force microscopy.
