# Supplementary material for: Extracellular matrix remodelling and stiffening contributes to tumorigenesis of salivary carcinoma ex pleomorphic adenoma——A study based on patient-derived organoids
Source: Cell Biosci. 2023 Jul 1;13:122. doi: 10.1186/s13578-023-01071-x (PMC10315042; doi:10.1186/s13578-023-01071-x)
Supplement: Supplementary file 1 — Additional file 1: Supplementary methods, tables and figures. [file 13578_2023_1071_MOESM1_ESM.docx]

**Supplementary data**

For “Extracellular matrix remodelling and stiffening contributes to carcinogenesis of salivary pleomorphic adenoma——A study based on patient-derived organoids”

**Supplementary Methods:**

**PA and CXPA Organoids Culture**

The culture medium was refreshed every 3-4 days. For passaging, the Matrigel was removed using a CultrexTM organoid harvesting solution (R&D System, USA), as instructed by the manufacturer. After centrifugation, organoids were pipetted up and down for several times. Dissociated organoids were centrifuged to remove the culture medium and seeded in Matrigel. Organoids were passaged at a 1:5-1:6 ratio every 2 weeks. For cryopreservation, they were dissociated from the Matrigel, dissociated into small clusters and frozen in Serum-free Cell Cryopreservation Medium (NCM, China).

**H&E, IHC staining, and WES Analysis of Organoids and their Parental Tumours**

Fixed tissue samples and agar pre-embedded organoids were dehydrated, paraffin-embedded and sectioned. H&E staining and immunostaining were performed on 4 μm-thick paraffin sections. For H&E staining, paraffin sections were deparaffinized in xylene and rehydrated using gradient ethanol concentrations. For immunohistochemical assays, paraffin slides were deparaffinized, rehydrated and subjected to antigen retrieval by incubation in EDTA solution (pH 8.0) for 20 min. The slides were incubated at 4°C overnight with the following primary antibodies: Ki67 (1:200; Maxim, Cat. MAB-0672, China), p63 (1:200; Maxim, Cat. MAB-0694, China), CK14 (1:200; Maxim; Cat. MAB-0169, China), CK7 (1:100; Maxim; Cat. Kit-0021, China), SMA (1:100; Gene; Cat. GM-085129, China), S100 (1:100; Maxim; Cat. Kit-0007, China), Calp (1:200; Maxim; Cat. MAB-0712, China), and CK19 (1:100; Maxim; Cat. Kit-0030, China). Then, slides were washed thrice using PBS and incubated with anti-rabbit or mouse IgG-HRP antibodies (1:200, DAKO, Denmark) for 30 mins at room temperature. Nuclei were counterstained with DAPI. Imaging was performed using a Leica LF200 microscope. The positive and negative stained markers were quantified in more than 3 fields for organoids and original tumours using the ImageJ software and analysed using GraphPad Prism 9.

**Identification and validation of the potential genes related to ECM remodelling and TWIST1 in PA and CXPA surgical specimens**

The SDT (4% SDS, 100 mM Tris-HCl, 1 mM DTT, pH 7.6) buffer was used for sample lysis and protein extraction. The amount of protein was quantified using the BCA Protein Assay Kit (Bio-Rad, USA). All protein samples were separately packed and stored at -80°C. Then, 20 µg of protein for each sample were respectively mixed with 5X loading buffer and boiled for 5 min at 100℃. The proteins were separated on 12.5% SDS-PAGE gel (constant current 14 mA, 90 min). Protein bands were visualized by Coomassie Blue R-250 staining. Protein digestion was performed using trypsin. The digested peptides of each sample were desalted on C18 Cartridges (Empore™ SPE Cartridges C18 (standard density), bed I.D. 7 mm, volume 3 ml, Sigma), concentrated by vacuum centrifugation and reconstituted in 40 µl of 0.1% (v/v) formic acid.

The LC-MS/MS analysis was performed on a Q Exactive mass spectrometer (Thermo Scientific) coupled to Easy nLC (Proxeon Biosystems, now Thermo Fisher Scientific) for 65 min. The peptides were loaded onto a reverse phase trap column (Thermo Scientific Acclaim PepMap100, 100 μm*2 cm, nanoViper C18) connected to the C18-reverse phase analytical column (Thermo Scientific Easy Column, 10 cm long, 75 μm inner diameter, 3μm resin) in buffer A (0.1% Formic acid), and separated with a linear gradient of buffer B (84% acetonitrile and 0.1% Formic acid) at a flow rate of 300 nl/min controlled by IntelliFlow technology. The mass spectrometer was operated in the positive ion mode. The MS data were acquired using a data-dependent top10 method dynamically choosing the most abundant precursor ions from the survey scan (300–1800 m/z) for HCD fragmentation. Automatic gain control (AGC) target was set to 3e6, while the maximum inject time was set to 10 ms. Dynamic exclusion duration was 40.0 s. Survey scans were acquired at a resolution of 70,000 at m/z 200 while the resolution for HCD spectra was set to 17,500 at m/z 200, and isolation width was 2 m/z. Normalized collision energy was 30 eV and the underfill ratio, which specifies the minimum percentage of the target value that is likely to be reached at maximum fill time, was defined as 0.1%. The instrument was run with peptide recognition mode enabled.

**Validation of simulated microenvironment stiffening in vitro influencing cell proliferation, migration and invasion of PA primary cells and CXPA cell line**

PA fresh tumour tissue was collected and cut into small fragments (about 1 mm3) and transferred into DMEM medium (Invitrogen, Carlsbad, CA, USA), with 10% fetal bovine serum (FBS, Gibco) and 1% penicillin-streptomycin. The tissue fragments were placed in a T-25 flask containing a 1.5 mL culture medium. The culture bottle was inverted overnight and gently turned over the second day, which was placed in a 37 ℃ incubator. After being grown for six days, cells were suspended with 0.25% trypsin and 0.02% EDTA (NCM, Suzhou, China) treatment and passaged with a 1:2 split ratio.

SM-AP1 cells were cultured in DMEM supplemented with 10% FBS and 1% penicillin-streptomycin.

# Supplementary Tables：

## Table S1 Baseline characteristics of the patients.

| **Case** | **Tumor** | **Age (Y)** | **Sex** | **Malignant components** | **Lymphatic metastasis** | **Distant metastasis** | **FISH**  **for**  ***HER-2*** | **FISH**  **for**  ***PLAG 1*** |
| --- | --- | --- | --- | --- | --- | --- | --- | --- |
| 1 | PA | 53 | Female |  | NO | NO | - | + |
| 2 | PA | 49 | Female |  | NO | NO | - | + |
| 3 | PA | 24 | Female |  | NO | NO | - | - |
| 4 | PA | 25 | Female |  | NO | NO | - | - |
| 5 | PA | 44 | Male |  | NO | NO | - | + |
| 6 | CXPA | 74 | Female | Salivary duct carcinoma | YES | NO | + | + |
| 7 | CXPA | 69 | Male | Salivary duct carcinoma | NO | NO | + | + |
| 8 | CXPA | 62 | Male | Salivary duct carcinoma | NO | NO | - | - |
| 9 | CXPA | 57 | Male | Salivary duct carcinoma | NO | NO | - | + |

| REAGENT | | | SOURCE | CONCENTRATION |
| --- | --- | --- | --- | --- |
| Chemicals and Recombinant Proteins | | | | |
| Advanced DMEM/F12 | | Invitrogen | |  |
| HEPES | Gibco | | | 1 × |
| GlutaMax | Gibco | | | 1 × |
| Primocin | Invivogen | | | 1 mg/ml |
| Penicillin-Strptomycin | Gibco | | | 10% |
| B-27 Supplement | Gibco | | | 1 × |
| Y-27632 | MCE | | | 10 μM |
| Nicotinamide | MCE | | | 10 mM |
| N-Acetyl-L-cysteine | MCE | | | 1.25 mM |
| Epidermal growth factor (EGF) | MCE | | | 50 ng/ml |
| Dexamethasone | MCE | | | 1 μM |
| Wnt3a | MCE | | | 500 ng/ml |
| R-spondin 1 | MCE | | | 0.1 μg/ml |
| Noggin | MCE | | | 0.1 μg/ml |
| N-2 Supplement | Gibco | | | 1 × |
| FGF2 | MCE | | | 5 ng/ml |

**Table S2 Culture medium for PA and CXPA organoids.**

**Table S3 Information about antibodies in IHC.**

| **REAGENT** | **SOURCE** | **DILUTION RATIO** | **IDENTIFIER** |
| --- | --- | --- | --- |
| **Antibodies** |  |  |  |
| DCN | Affnity, USA | 1:50 | DF6543 |
| COL1A1 | Abcam, UK | 1:1500 | Ab138492 |
| IGFBP5 | Affinity, USA | 1:50 | DF6574 |
| TWIST1 | Abclonal,China | 1:1000 | A15596 |

**Supplementary Figures:**

**
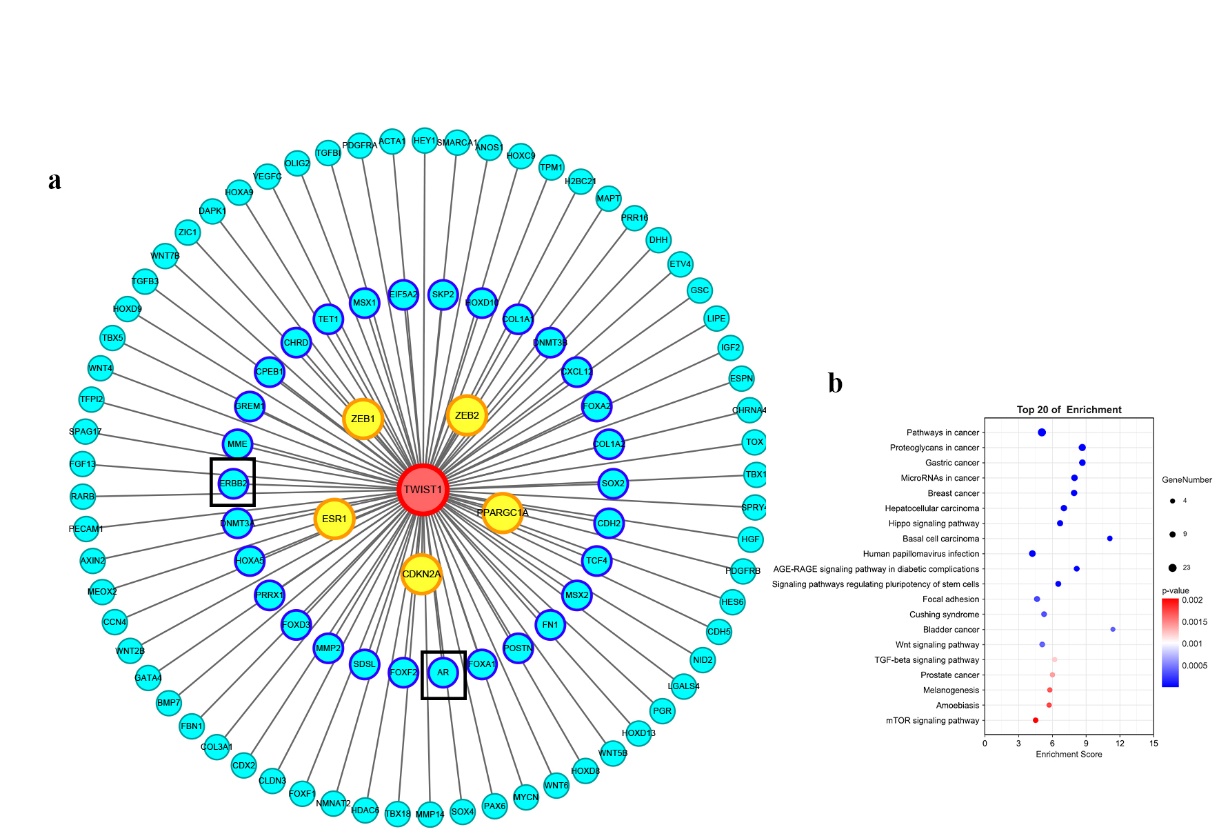
**

**Fig. S1 PPI analysis and KEGG enrichment analysis.** a. The PPI network of the top 100 genes co-expressed with TWIST1 in CXPA organoids. b. KEGG enrichment results of the top 100 differentially expressed genes co-expressed with TWIST1 in CXPA organoids.


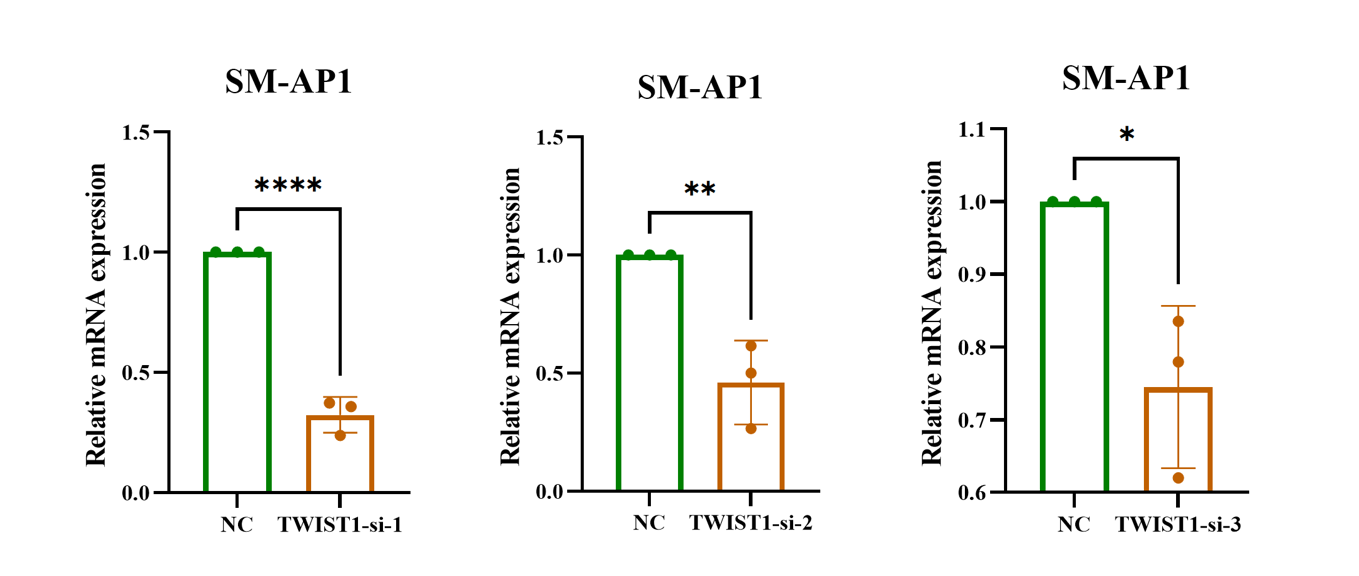


**Fig. S2 Relative mRNA expression after knocking down TWIST1 in SM-AP1 cells.**
